# Concentrations of remdesivir and GS-441524 in human milk from lactating individuals diagnosed with COVID-19

**DOI:** 10.1038/s41390-024-03053-2

**Published:** 2024-02-12

**Authors:** Kerri Bertrand, Yadira Sepulveda, Benjamin J. Spiegel, Brookie M. Best, Raymond Suhandynata, Steven Rossi, Christina D. Chambers, Jeremiah D. Momper

**Affiliations:** 1 Department of Pediatrics, University of California, San Diego, La Jolla, CA 92093 USA; 2 Skaggs School of Pharmacy and Pharmaceutical Sciences, University of California, San Diego, La Jolla, CA 92093 USA; 3 Herbert Wertheim School of Public Health and Human Longevity Science, University of California, San Diego, La Jolla, CA 92093 USA

## Abstract

- Findings from this study provide further reassuring evidence that infant exposure through human milk received from lactating individuals who require treatment with remdesivir is negligible.

## Background

Pregnant and postpartum individuals are at high risk for severe illness from COVID-19.^1^ Remdesivir is a SARS-CoV-2 nucleotide analog RNA polymerase inhibitor and the first drug approved by the U.S. Food and Drug Administration (FDA) for the treatment of COVID-19.^2^ Remdesivir is rapidly converted by plasma and tissue esterases to an alanine intermediate (GS-704277) which is further metabolized to the pharmacologically active nucleoside analog monophosphate metabolite (GS-441524).^2^ Although remdesivir has a short half-life (0.90–0.96 h), the half-life of GS-441524 is approximately 27 h in healthy, non-pregnant adults.^2^

Lactating individuals undergoing treatment with remdesivir are typically advised not to breastfeed or to breastfeed with careful infant monitoring due to a paucity of data on the excretion of remdesivir and its metabolite GS-441524 in human milk.^3^ However, breastfeeding while infected with SARS-CoV-2 is considered safe and is recommended since antibodies to SARS-CoV-2 are transmitted through human milk.^4,5^ To date, there is a single case report published in the literature on remdesivir treatment in a lactating individual.^6^ Wada et al. reported low levels of remdesivir and GS-441524 in human milk and estimated a relative infant dose (RID) of 0.007%.^6^ The objective of this study was to add to the very limited available data by measuring the concentration of remdesivir and GS-441524 in human milk from lactating individuals receiving remdesivir for the treatment of COVID-19.

## Methods

### Study design and population

Between March 2020 and May 2020, three lactating persons residing in the U.S. who reported the use of remdesivir consented to participation in the Human Milk Research Biorepository (HMB) at the University of California, San Diego (UCSD). Participants provided written consent and completed an interview on demographics, maternal and child health history, and details regarding prescription medications and other lifestyle exposures for the 14 days prior to milk sample collection. Participants also answered questions about their COVID-19 illness, testing, symptoms, and treatments. Human milk samples were collected using a standardized protocol that has previously been described.^7^ Additionally, a fourth participant provided written consent and enrolled in the IMPAACT 2032 study (Pharmacokinetics and Safety of Remdesivir for Treatment of COVID-19 in Pregnant and Non-Pregnant Women in the United States) and collected one milk sample, which was included in this analysis.^8,9^ All samples were stored at –80 °C within 3 h after collection until the time of transfer to the analytical laboratory. The UCSD Institutional Review Board (IRB) approved the HMB study (IRB #130658). IMPAACT 2032 was approved by the Johns Hopkins Medicine IRB serving as a single Institutional Review Board.

### Determination of remdesivir and GS-441524 in human milk

Calibrators and quality control samples were prepared from standard reference material: Remdesivir (Chemical Formula: C27H35N6O8P) and GS-441524 (Chemical Formula: C12H13N5O4) (Gilead). Internal standards (IS) were prepared from standard reference material: [^13^C3]-GS-5734 (GS-289143) and [^13^C3]-GS-441524 (GS-828840) (Gilead). Calibration standards were prepared in human milk and used to generate a calibration curve using linear regression to plot the peak area ratio versus concentration with 1/*x* weighting (*r*^2^ ≥ 0.99), over the full analytically reportable range (ARR). The ARR for this assay was 100–800 ng/mL for remdesivir and 100–1000 ng/mL for GS-441524.

Concentrations of remdesivir and GS-441524 in human breastmilk were determined by ultra-high-performance liquid chromatography (UHPLC) and multiple reactions monitoring mass spectrometry (MRM) (Agilent Infinity 1290 and AB SCIEX 6500 + QTRAP). MRM was performed in positive electrospray ionization mode (ESI) using an HSS T3 UPLC column (2.1 × 50 mm, 1.8 µm, Waters, Milford). Quality control (QC) samples were prepared at 300 ng/mL and 600 ng/mL in human milk for both remdesivir and GS-441524.

Remdesivir and GS-441524 were extracted from 50 µL of human milk spiked with 100 µL of IS. Proteins were precipitated using 600 µL of 50% MeOH: 50% ACN and vortexed for 30 seconds before centrifugation at 21,000 RCF for 10 minutes at 4 °C. 300 µL of the supernatant was subsequently diluted with 600 µL of 18.2 MΩ-cm H_2_O and 8 µL was injected via the autosampler for LC-MRM analysis. The method was validated in human milk by establishing the accuracy and precision of 3 sets of calibration curves and QC samples over 3 days. The acceptability criteria for accuracy (within the run and between runs) was ±15% of nominal concentrations except ±20% at the lower limit of quantification (LLOQ). The acceptability criteria for precision (within-run and between runs) was ±15% coefficient of variation (CV), except ± 20% CV at LLOQ.

The stability of remdesivir and GS-441524 in human milk was evaluated under various storage conditions selected to approximate storage and handling encountered within the study. Remdesivir and GS-441524 were spiked into blank human milk at a nominal concentration of 500 ng/mL and evaluated at room temperature (1 and 4 h), 4 °C (1, 4, and 24 h), –20 °C (1 and 2 weeks), and –80 °C (1 and 2 weeks).

### Estimation of relative infant dose

The RID of remdesivir and GS-441524 was calculated in order to estimate potential infant exposure.^10^ The daily infant dose (mg/kg) was determined by multiplying an average milk intake of 150 mL/kg/day by the maximum concentration of each analyte observed in milk. The RID was then calculated by dividing the daily infant dose by the daily maternal dose (mg/kg). A maternal weight of 70 kg was used.

## Results

**Fig. 1:**
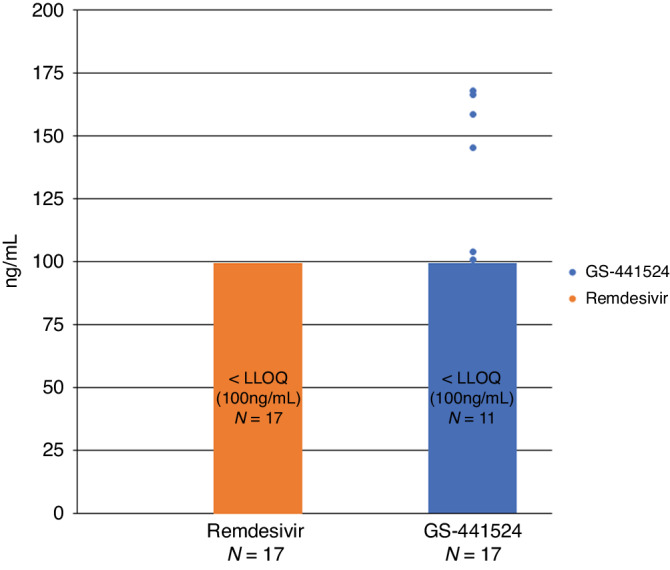
Remdesivir and GS-441524 concentrations determined in human human milk as measured by isotope dilution LC-MS/MS. The lower limit of quantification (LLOQ) of the assay for both parent and metabolite was set at 100 ng/mL in human milk. Orange and blue bars represent concentrations less than the LLOQ for each analyte. The total number of specimens tested is indicated on the *x*-axis below each individual analyte, while the number of specimens that were measured to be <LLOQ for remdesivir (orange) and GS-441524 (blue) are indicated within each bar. Measured values above the LLOQ for each individual specimen are represented by points on the graph corresponding to their measured concentrations in ng/mL (*y*-axis).

Seventeen human milk samples from four lactating individuals were analyzed (Fig. 1). Characteristics of the participants are shown in Table 1. All four participants were hospitalized and receiving treatment with remdesivir for COVID-19 at the time of enrollment. Participants received remdesivir as an intravenous infusion with a single 200 mg loading dose on Day 1 followed by once-daily maintenance doses of 100 mg. Thirteen of the milk samples were collected between 4 h and 61 days following an administration of remdesivir. The sample collection time relative to remdesivir dosing was unknown in 4 of 17 samples. For remdesivir, all 17 (100%) of the samples were below the LLOQ(100 ng/mL). For GS-441524, 11 of 17 (65%) of the samples were below the LLOQ (100 ng/mL). Of the remaining 6 (37.5%) samples from 1 unique participant, the median (interquartile range) GS-441524 concentration was 152 ng/mL (103–168 ng/mL). The maximum observed GS-441524 concentration in human milk was 168 ng/mL. Assuming a 150 mL/kg/day infant milk intake, the estimated RID of remdesivir was <1% and the estimated RID of GS-441524 was <5%. No degradation was observed for remdesivir and GS-441524 in human milk under the storage conditions evaluated.

**Table 1:** Selected characteristics of maternal and infant participants with exposure to remdesivir who enrolled in mommy’s milk, The human milk biorepository between March and May 2020, *N* = 3.

Characteristic,	*N* = 3^a^, *n*^1^ (%)
Maternal age	*n*^1^ = 3
Median (range)	40.03 (38.10–40.86)
Maternal ethnicity	*n*^1^ = 3
Hispanic	0 (0.00)
Non-Hispanic	3 (100.00)
Race	*n*^1^ = 3
White	1 (33.33)
Asian	1 (33.33)
Native American	1 (33.33)
BMI	*n*^1^ = 2
18.5–24.99	1 (50.00)
>30	1 (50.00)
Other prescription medications	*n*^1^ = 2
Yes	1 (50.00)
No	1 (50.00)
Alcohol use	*n*^1^ = 2
No	2 (100.00)
Tobacco use	*n*^1^ = 2
No	2 (100.00)
Recreational drug use	*n*^1^ = 2
No	2 (100.00)
Infant age (months)	*n*^1^ = 3
median (range)	0.23 (0.06-2.99)
Infant sex	*n*^1^ = 2
Male	2 (100.00)
Infant feeding type	*n*^1^ = 3
Exclusive breastfeeding without solid foods	2 (66.67)
Supplemented with formula or donor milk without solid foods	1 (33.33)
Infant adverse reactions^b^	*n*^1^ = 3
No	3 (100.00)
Milk expression method	*n*^1^ = 3
Electric pump	3 (100.00)
Full milk expression	*n*^1^ = 3
Yes	3 (100.00)

^a^Information from the fourth study subject who was enrolled in IMPACT 2023 was not included.

^b^A standardized checklist of infant adverse reactions

## Discussion

Remdesivir and GS-441524 concentrations in human milk were below the level of clinical concern in the samples evaluated in this study. The concentration of remdesivir was below the LLOQ (100 ng/mL) in all 17 samples tested. The estimated RID of remdesivir was <1%, which is considerably below the acceptability level of 10% for presumed safety in infants.^11–13^ Furthermore, as remdesivir is administered intravenously due to poor oral bioavailability, systemic infant exposure via milk consumption is expected to be negligible.

The metabolite GS-441524 was quantifiable in only 6 of 17 samples tested with a maximum concentration of 168 ng/mL. All of the 6 milk samples with quantifiable GS-441524 concentrations were collected between 4 and 12 h following administration of remdesivir. The RID of GS-441524 could not be reliably estimated because the maternal dose of the metabolite is undefined. With a 150 mL/kg/day infant milk intake at the maximum observed GS-441524 milk concentration of 168 ng/mL, the weight-based infant dose of GS-441524 would be 0.0252 mg/kg/day. In adults, 48.6% of the remdesivir dose is recovered in urine as GS-441524. Using these values, the RID of GS-441524 via human milk was < 5% of the weight-adjusted adult dose. The present results are similar to those of Wada et al. who reported that remdesivir was either not detected or insignificant in 4 human milk samples from a single lactating woman, with an estimated RID of 0.007%.^6^ In addition, GS-441524 milk concentrations were similar in both studies.^6^

Remdesivir is approved by the FDA for the treatment of COVID-19 in pediatric patients 28 days of age and older and weighing at least 3 kg at a loading dose of 5 mg/kg and a once-daily maintenance dose of 2.5 mg/kg. No serious adverse reactions have been observed in clinical trials of remdesivir across these dose levels in pediatric patients as young as 28 days.^14^

This study had several limitations. Samples were collected under different conditions; not all breastmilk collections were directly observed. We relied on maternal reports of remdesivir exposure; however, the 3 HMB participants completed a 14-day recall guided by trained study staff who prompted for specific daily use with the aid of a calendar. In addition, samples were collected over a wide time range following a dose of remdesivir and the sample time relative to dosing was unknown for 4 samples. It is possible that remdesivir and GS-441524 concentrations in milk may be higher if samples are obtained within approximately 1.5 h after the end of the infusion, as this time corresponds to the highest remdesivir and GS-441525 concentrations in maternal plasma. Finally, infant plasma samples were not collected; therefore, infant exposure could not be directly assessed.

In conclusion, these data further confirm that infant exposure to remdesivir and GS-441524 through human milk ingestion would be negligible.

## Data Availability

The dataset generated during and/or analyzed during the current study is available from the corresponding author upon reasonable request.

## References

[1] Hollier, L. *Covid-19, Pregnancy, Childbirth, and Breastfeeding: Answers from Ob-Gyns* https://www.acog.org/womens-health/faqs/coronavirus-covid-19-pregnancy-and-breastfeeding#:~:text=Pregnant%20and%20postpartum%20women%20have,prenatal%20and%20postpartum%20care%20visits (2022).

[2] Jorgensen, S. C. J., Kebriaei, R. & Dresser, L. D. Remdesivir: review of pharmacology, pre-clinical data, and emerging clinical experience for Covid-19. *Pharmacotherapy: J. Hum. Pharmacol. Drug Ther.* **40**, 659–671 (2020). 10.1002/phar.2429 PMC7283864 32446287

[3] Medicine, N. L. O. *Drugs and Lactation Database (LactMed) Vol. 2022* (National Institute of Child Health and Human Development, 2006).

[4] Pace, R. M. et al. Covid-19 and human milk: SARS-CoV-2, antibodies, and neutralizing capacity. *medRxiv*10.1101/2020.09.16.20196071 (2020).

[5] WHO. *Breastfeeding and Covid-19: Scientific Brief* (WHO, 2020).

[6] Wada, Y. S. et al. Remdesivir and human milk: a case study. *J. Hum. Lact.* **38**, 248–251 (2022). 35189734 10.1177/08903344221076539

[7] Bandoli, G., Bertrand, K., Saoor, M. & Chambers, C. D. The design and mechanics of an accessible human milk research biorepository. *Breastfeed. Med.* **15**, 155–162 (2020). 31985264 10.1089/bfm.2019.0277 PMC7133453

[8] IMPAACT. *Impaact 2032 Protocol Version 2.0 with Cm #1 and Corrected Cm #2*, https://www.impaactnetwork.org/studies/impaact2032 (IMPAACT, 2021).

[9] Brooks K, et al. 268. *Abstracts from CROI 2022 Conference on Retroviruses and Opportunistic Infections* (2022).

[10] Anderson, P. O. & Momper, J. D. Clinical lactation studies and the role of pharmacokinetic modeling and simulation in predicting drug exposures in breastfed infants. *J. Pharmacokinet. Pharmacodyn.* **47**, 295–304 (2020). 32034606 10.1007/s10928-020-09676-2

[11] Hoddinott, P., Tappin, D. & Wright, C. Breast feeding. *BMJ* **336**, 881–887 (2008). 18420694 10.1136/bmj.39521.566296.BE PMC2323059

[12] Rowe, H., Baker, T. & Hale, T. W. Maternal medication, drug use, and breastfeeding. *Child Adolesc. Psychiatr. Clin. N. Am.* **24**, 1–20 (2015). 25455573 10.1016/j.chc.2014.09.005

[13] Sachs, H. C., Committee On, D. The transfer of drugs and therapeutics into human breast milk: an update on selected topics. *Pediatrics* **132**, e796–e809 (2013). 23979084 10.1542/peds.2013-1985

[14] Administration, U. S. F. a. D. *Highlights of Prescribing Information* https://www.accessdata.fda.gov/drugsatfda_docs/label/2023/214787s024lbl.pdf (Administration, U. S. F. a. D, 2023).

